# Integrating experimental and literature protein-protein interaction data for protein complex prediction

**DOI:** 10.1186/1471-2164-16-S2-S4

**Published:** 2015-01-21

**Authors:** Yijia Zhang, Hongfei Lin, Zhihao Yang, Jian Wang

**Affiliations:** 1 College of Computer Science and Technology, Dalian University of Technology, Dalian, Liaoning, China

## Abstract

**Background:**

Accurate determination of protein complexes is crucial for understanding cellular organization and function. High-throughput experimental techniques have generated a large amount of protein-protein interaction (PPI) data, allowing prediction of protein complexes from PPI networks. However, the high-throughput data often includes false positives and false negatives, making accurate prediction of protein complexes difficult.

**Method:**

The biomedical literature contains large quantities of PPI data that, along with high-throughput experimental PPI data, are valuable for protein complex prediction. In this study, we employ a natural language processing technique to extract PPI data from the biomedical literature. This data is subsequently integrated with high-throughput PPI and gene ontology data by constructing attributed PPI networks, and a novel method for predicting protein complexes from the attributed PPI networks is proposed. This method allows calculation of the relative contribution of high-throughput and biomedical literature PPI data.

**Results:**

Many well-characterized protein complexes are accurately predicted by this method when apply to two different yeast PPI datasets. The results show that (i) biomedical literature PPI data can effectively improve the performance of protein complex prediction; (ii) our method makes good use of high-throughput and biomedical literature PPI data along with gene ontology data to achieve state-of-the-art protein complex prediction capabilities.

## Background

Protein complexes are formed from two or more associated polypeptide chains, and accurate determination of protein complexes is of great importance for understanding cellular organization and function. Many proteins are only functional after assembly into protein complexes. Even in the relatively simple model organism Saccharomyces cerevisiae, protein complexes include many subunits that assemble and function in a coherent fashion. A key task of system biology is to understand proteins and their interactions in terms of protein complexes [1].

Recent advances in high-throughput experimental techniques such as yeast two-hybrid and mass spectrometry have generated a large amount of protein-protein interaction (PPI) data for numerous organisms [2,3]. These high-throughput PPI data facilitate the development and testing of computational methods for protein complex prediction. The molecular complex detection (MCODE) algorithm proposed by Bader and Hogue [4] was one of the first computational methods reported. The Markov clustering algorithm [5] was also applied to predict protein complexes by simulating random walks within PPI networks. Adamcsek et al. developed the CFinder tool [6] that found functional modules in PPI networks using the clique percolation method [7] to detect k-clique percolation clusters. Liu et al. proposed a clustering method based on maximal cliques (CMC) to detect protein complexes [8]. Wu et al. developed the COACH algorithm [9] based on core-attachment structural features [10]. COACH initially identifies protein-complex cores at the heart of protein complexes, then attaches other proteins to these cores. Since proteins may have multiple functions, they may belong to more than one protein complex. Nepusz et al. proposed the ClusterONE algorithm [11] which detected overlapping protein complexes in PPI networks.

One major problem with high-throughput experimental PPI data is the high incidence of both false positives and false negatives [12]. Computational methods that only use high-throughput PPI data do not generally predict protein complexes accurately. This situation is improved if gene expression and gene ontology (GO) data are included. Feng et al. used microarray data to weight PPI networks, and this markedly improved the initial binary PPI networks [13]. Zhang et al. proposed the COAN algorithm based on ontology augmentation networks constructed with high-throughput PPI and GO annotation data. COAN takes into account the topological structure of the PPI network, as well as similarities in GO annotations [14].

The biomedical literature contain a large amount of potentially valuable PPI data that can be used to further improve protein complex prediction algorithms. In this study, we attempt to use this resource by first employing a natural language processing technique to extract PPI data from the biomedical literature. This is then integrated with high-throughput PPI and GO data by constructing attributed PPI networks that can be used for protein complex prediction. This novel approach automatically calculates the relative contributions of high-throughput and biomedical literature PPI data. The method is compared with current protein complex prediction tools. The advantages of the method, potential applications and improvements are discussed.

## Methods

### Extracting PPI data from the literature

The biomedical literature contains lots of potentially valuable PPI data, and extraction of this data is an important research topic in the field of biomedical natural language processing [15,16].

**Figure 1:**
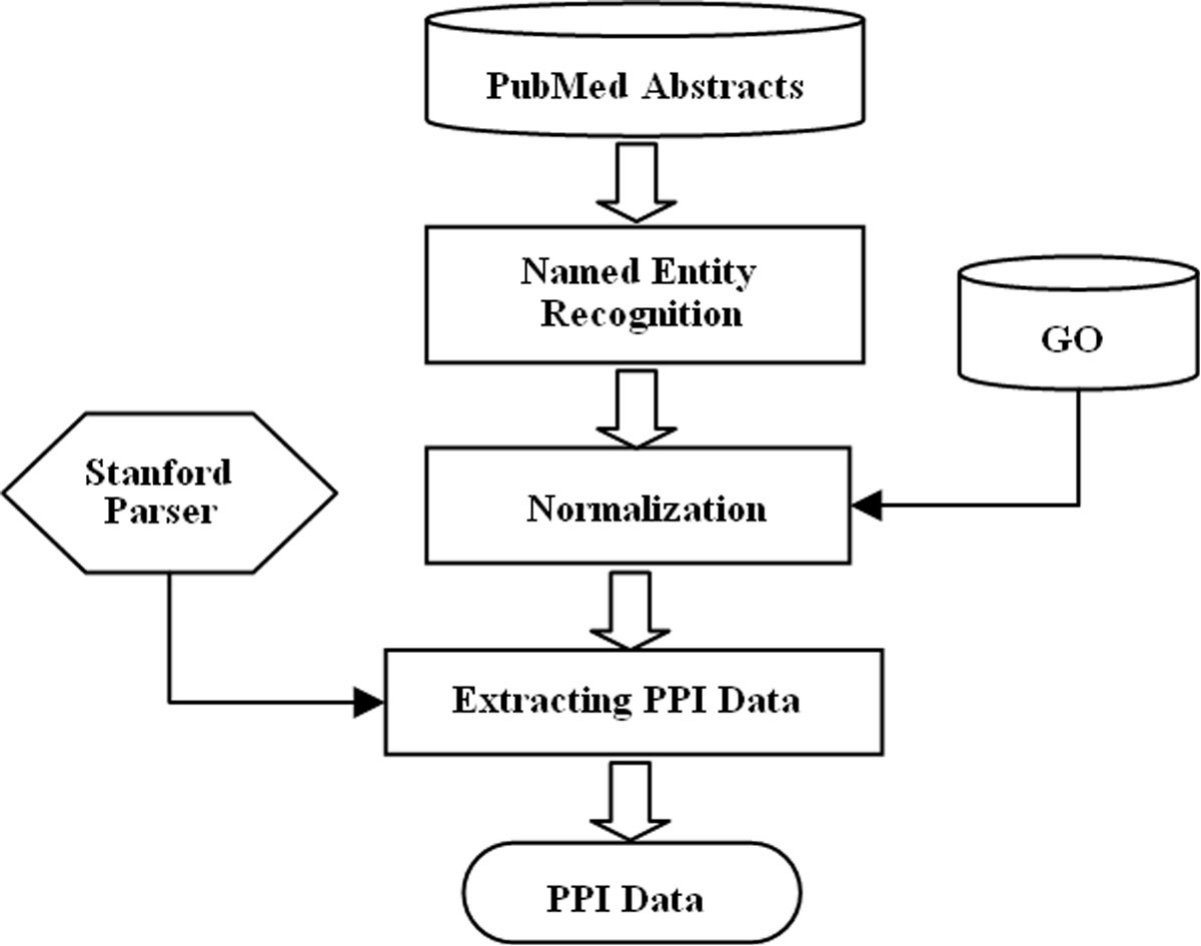
**Workflow for extracting PPI data from PubMed abstracts**.

Our method of extracting PPI data consists of three phases: (i) named entity recognition (NER); (ii) normalization; (iii) extracting PPI data (Figure 1). NER aims to identify protein names in the biomedical literature. In our method, we use the FCG model [17] which is a semi-supervised learning strategy. FCG involves learning a novel feature representation from the co-occurrence of class-distinguishing features (CDFs) and example-distinguishing features (EDFs). CDFs and EDFs refer to strong indicators for classes and for examples, respectively. Their co-occurrence in large unlabeled datasets captures information that can not be obtained from labeled training data due to data sparseness.

Protein name normalization is used to determine the unique protein identifiers mentioned in the literature, linking these entities to biological databases. It is difficult to choose between ambiguous protein names based on context and short textual descriptions. We decide to adopt a disambiguation method [16] based on extended semantic similarity, which enriches gene descriptions in databases with information extracted from GO and PubMed abstracts. This allow us to exploit context and extend semantic information.

In the three phases, we extract PPI data from biomedical literature based on NER and normalization. Pattern-based methods is an established methodology for PPI extraction that usually uses defined lexical patterns and retrieves text segments that match the patterns. Because this approach is too rigid to capture semantic/syntactic paraphrases or distant relationships, such pattern-based methods always suffer from low recall rates. Instead, publicly accessible annotated PPI corpora such as GENIA [18] and AImed [19] allow automatic extraction of PPI data using machine learning methods. Recent studies [15,16] have established the power of machine learning methods, which handle PPI extraction as a classification problem. The major challenge is in supplying the learner with the semantic/syntactic information-containing features in order to distinguish between interactions and non-interactions.

Initial filtering of sentences that contain at least two protein entities is performed, and using the Stanford lexical parser generates syntactic information. At this stage, including a dependency graph or syntactic parse tree of candidate sentences maximizes the chances of efficient and accurate data extraction. However, syntactic information such as syntactic parse tree is not easily represented by flat features. Kernel methods can efficiently compute the similarity between structural data in a recursive manner without explicitly enumerating with feature vectors, avoiding complex feature construction and selection processes. We use the hash subgraph pairwise (HSP) kernel method to extract PPI data from biomedical literature, as proposed in our previous work [15]. HSP kernel methods compute hierarchical hash labels of syntactic structure based on hash operations in a linear time. The hierarchical labels consist of basic labels and hash labels for each node of dependency graph or syntactic parse tree. Basic labels represent the lexical features and hash labels represent the complex syntactic features. In our previous work, we have demonstrated the advantages of the HSP kernel method over other popular machine learning methods [15].

### Construction of attributed PPI networks

**Figure 2:**
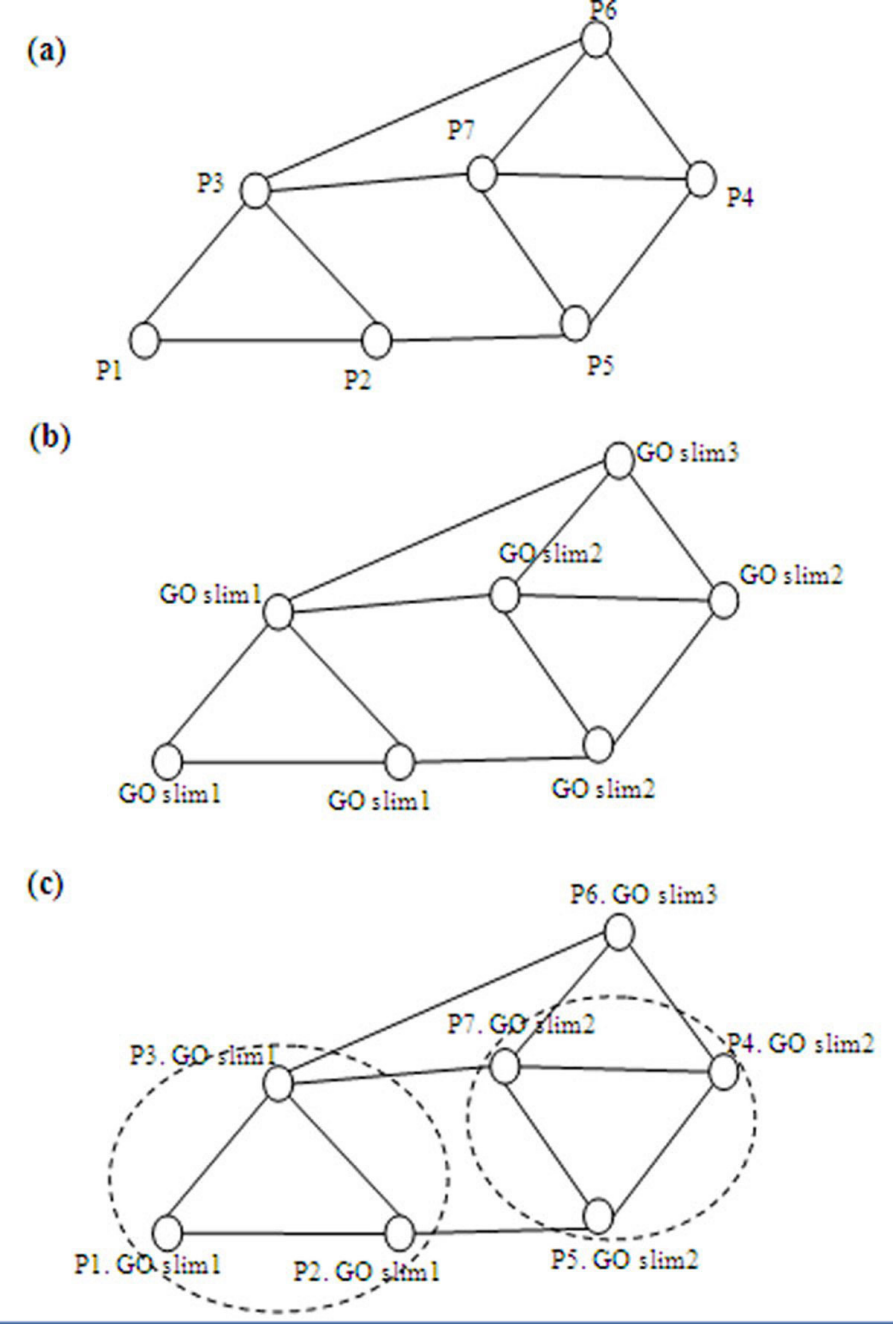
**Example of a protein complex prediction network**. (a) A PPI network of eight proteins. (b) The PPI network is annotated using GO slims. (c) Prediction of two protein complexes in the PPI network based on structural and GO annotation similarities.

Most computational methods for complex prediction are clearly limited by the poor quality of high-throughput PPI data. Further improvements for complex prediction can be obtained by integrating biomedical literature PPI data. GO is another useful resource for protein complex prediction, which is currently one of the most comprehensive ontology databases in the bioinformatics community [20]. GO aims to standardize the annotation of genes and gene products across species and provides a controlled vocabulary of terms for describing gene product biological properties. Due to the inherent biological properties of protein complexes [10], GO provides valuable PPI data for protein complex prediction. An example of a simple PPI network in which a vertex represents a protein and an edge represents the interaction between two proteins is shown in Figure 2(a). Due to the presence of noise and the complex connectivity of PPI data, it is hard to predict protein complexes from this type of network. Figure 2c shows that two protein complexes can be predicted reasonably accurately when the PPI network is annotated by GO slims (Figure 2b). Therefore, an accurate method for protein complex prediction should generate similar clusters based on topological structure and GO annotation. In this study, we integrate high-throughput experimental PPI data, biomedical literature PPI data, and GO to predict protein complexes using attributed PPI networks.

We define an attributed PPI network as a 6-tuple *G *= (*V*, *E*, *A_v_*, *A_e_*, *F_v_*, *F_e_*) where *V *is the set of protein vertices, *E *is the set of PPIs, *A_v _*= {*GS*_1_, *GS*_2_,...*GS_n_*} is the set of GO slim attributes for protein vertices, and *F_v _*is a function that returns the set of GO slim attributes of a protein vertex. Each protein vertex *pi *in *V *has a set of GO slim attributes *F_v_*(*p_i_*) = {*GS*_*i*1_,*GS*_*i*2_,...,*GS*_*im*_}, where *m *= |*F*_*v*_(*Pi*)| and *Fv*(*Pi*) ⊆ *A*_*v*_. Likewise, *A*_*e *_= {*T*_1_, *T*_2_,...*T_s_*} is the set of type attributes for PPIs, and *F_e _*is a function that returns the set of type attributes of a PPI. Each PPI *e_i _*in *E *has a set of type attributes *F_e_*(*e_i_*) = {*T*_*i*1_,*T*_*i*2_,...,*T*_*ir*_}, where *r *= |*F*_*e*_(*e*_*i*_)|, *F*_*e*_(*e*_*i*_) ≠ Ø and *Fe*(*ei*) ⊆ *Ae*. In this study, the type attributes of PPIs included high-throughput type and biomedical literature type (*A*_*e *_= {*T*_1_, *T*_2_}).

**Figure 3:**
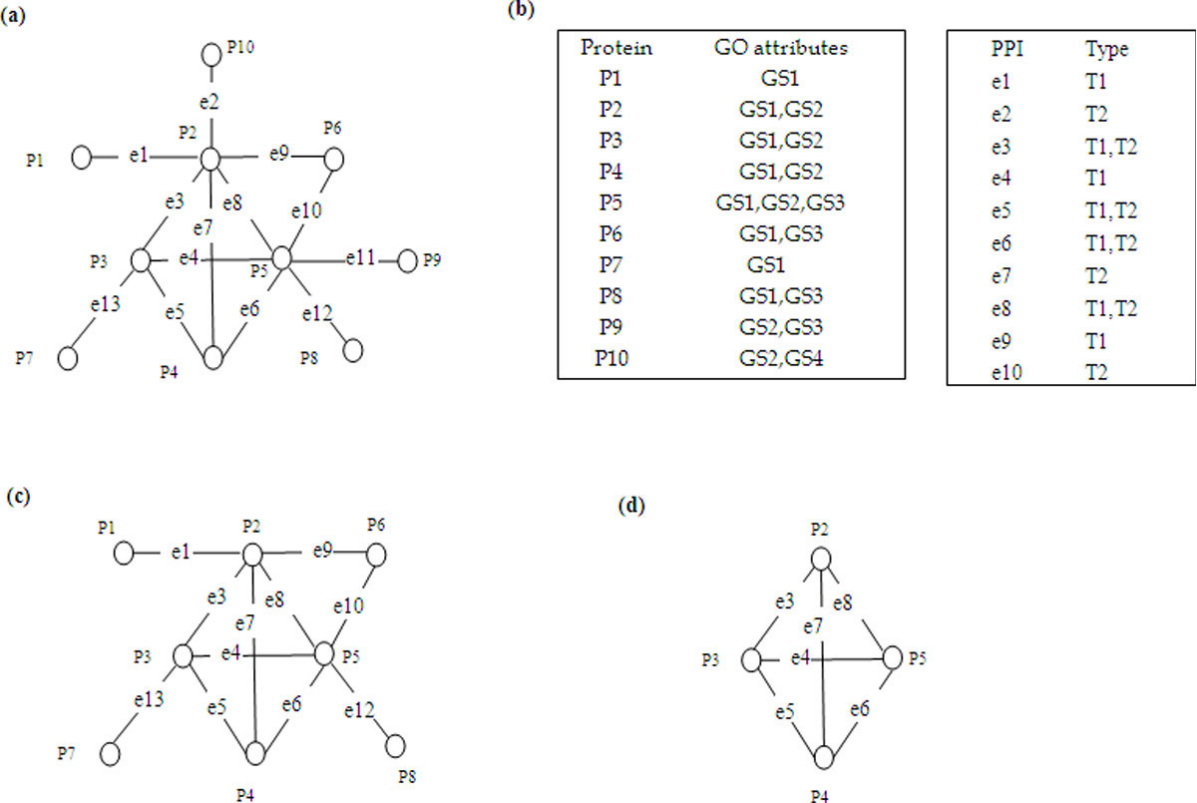
**Example of an attributed PPI network**. (a) an attributed PPI network. (b) GO slim attributes of protein vertices and type attributes of the PPIs. T1: high-throughput type; T2: biomedical literature type. (c) and (d) are the subgraphs induced by {GS1} and {GS1,GS2} respectively.

Figure 3a shows an example of an attributed PPI network. The GO slim attributes of protein vertices and type attributes of PPI data are given in Figure 3b. It can be seen that each protein vertex has a GO slim attribute set and each edge has a type attribute set. For instance, *P*_2 _has two GO slim attributes (GS1 and GS2), and *e*_3 _has two type attributes (T1, high-throughput type; T2 biomedical literature type). Given the set of GO slim attributes *A_v_*, we define an attribute set *S *as a subset of *Av *(*S *⊆ *Av*). Moreover, we denote by *V*|(*S*) ⊆ *V *the vertex set induced by *S *(i.e., *V *(*S*) = {*P_i _*∈ *V*|*S *⊆ *F_v_*(*P_i_*)}) and by *E*(*S*) ⊆ *E *as the edge set induced by *S *(i.e., *E*(*S*) = {(*P_i_*, *P_j_*) ∈ *E|P_i_*, *P_j _*∈ *V *(*S*)}). The subgraph *G*(*S*), induced by *S*, is the pair (*V *(*S*), *E*(*S*)). Figure 3c,d are the subgraphs induced by the attribute set{*GS*_1_}and{*GS*_1_,*GS*_2_}, respectively.

### Ontology correlated clique score

Definition 1 - Ontology correlated clique. Given a protein vertex set *C *and an edge set *E_c _*in the induced subgraph *G*(*S*) (*C *⊆ *V *(*S*), *E_c _*= {(*P_i_*, *P_j_) *∈ *E*(*S*)|*P=*, *P_j _*∈ *C*}), an ontology correlated clique is a pair ((*C*, *E_c_*), *S*), such that for each protein vertex *Pi *in *C*, the degree of *P_i _*is |*C*| −1. *S *is the common ontology attribute set of *C*.

**Figure 4:**
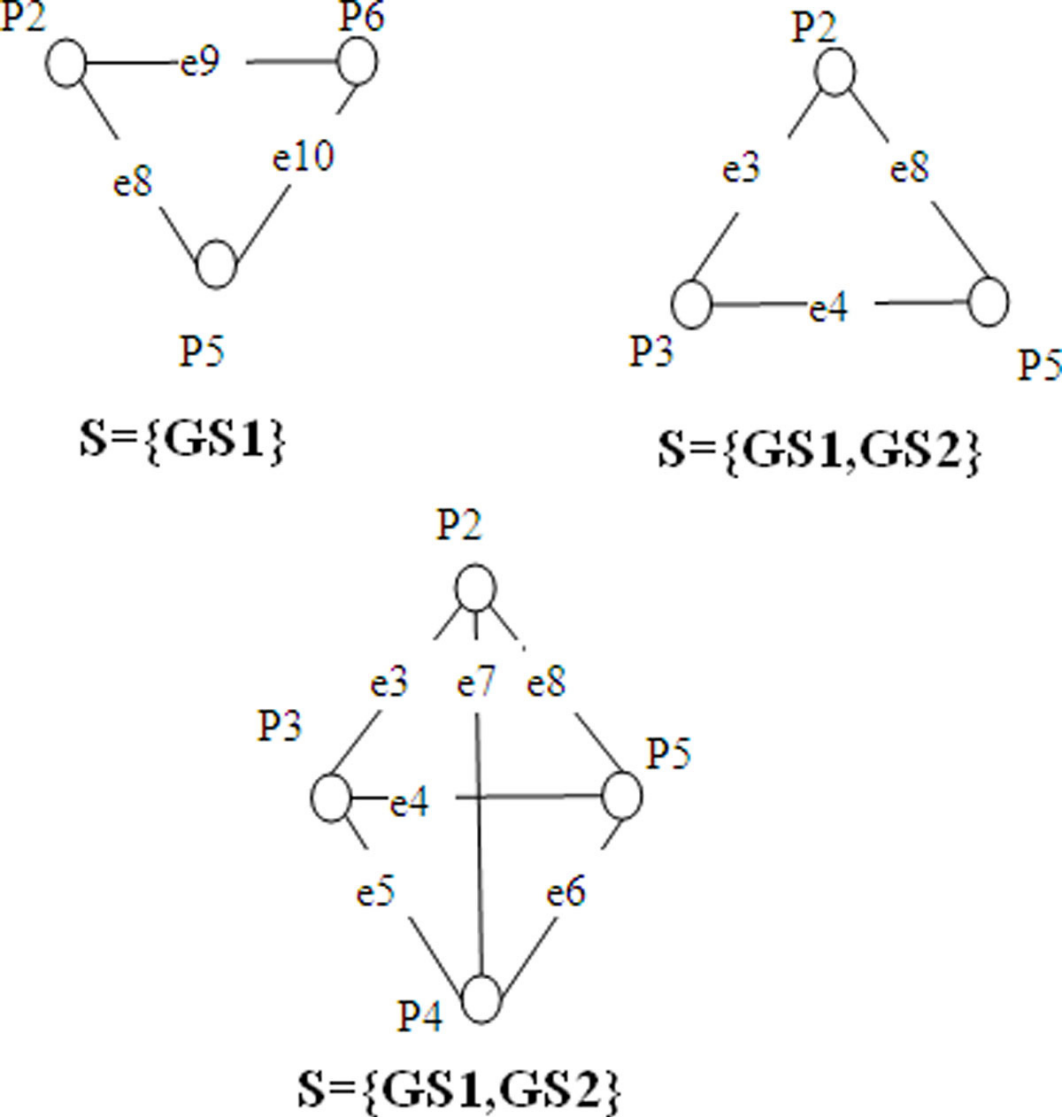
**Examples of ontology correlated cliques**.

In general, we can mine many Ontology correlated cliques with different common ontology attribute sets in an attributed PPI network. Figure 4 shows three ontology correlated cliques of the attributed PPI network in Figure 3.

Definition 2 - Structural correlated function *η*. Given an ontology slim attribute set *S*, the structural correlation of *S*, *η*(*S*), is given as:

(1) $$\eta \left(S\right)=\frac{\left|K_{s}\right|}{\left|V\left(S\right)\right|}$$

where *K_s _*is the set of vertices in ontology correlated cliques in *G*(*S*). In this study, we only considered cliques of three or more.

A correlation function can be used to measure the dependence between ontology attribute set *S *and the density of the associated vertices [21]. This indicates how likely *S *is to be part of a clique. The larger the structural correlation function *η*(*S*), the more valuable the ontology attribute set *S*. In Figure 3, *K*_{*GS *1} _= {*P*_2_, *P*_3_, *P*_4_, *P*_5_, *P*_6_}, *K*_{*GS *3} _= {} and *K *_{*GS *1, *GS *2} _= {*P*_2_, *P*_3_, *P*_4_, *P*_5_}. Thus the corresponding values of *η*({*GS*_1_}),*η*({*GS*_3_}) and *η*({*GS*_1_, *GS*_2_}) are 0.625, 0 and 1, respectively. Therefore, the protein vertices annotated by ontology attribute set{*GS*_1_, *GS*_2_} are more likely to be part of a protein complex than those annotated by{*GS*_1_}or{*GS*_3_}.

Definition 3 - Attributed network density. Given an attributed network *G *= (*V*, *E*, *A_v_*, *A_e_*, *F_v_*, *F_e_*), the density of *G*, *Density*(*G*), is given as:

(2) $$Weight\left(e_{i}\right)=\sum_{T_{j}\in F_{e}\left(e_{i}\right)}wj$$

(3) $$Density\left(G\right)=\frac{2\cdot \sum_{ei\in E}Weight\left(e_{i}\right)}{\left|V\right|\cdot \left|V-1\right|}$$

Since high-throughput PPI data and biomedical literature PPI data may differ in importance for protein complex prediction, we assign a weight to each type attribute to model their relative contributions. In equation (2), *w_j _*denotes the weight of the contribution of type attribute *T_j _*of the PPIs, and *weight*(*e_i_*) is the weight of *ei*. Moreover, the edges of attributed networks may have different weights due to their different type attributes. For example, if we set *w*_1 _= 0.6 and *w*_2 _= 0.4, *weight*(*e*_1_), *weight*(*e*_2_) and *weight*(*e*_3_) are 0.6, 0.4 and 1, respectively (Figure 3). This indicates that *e*_3 _makes a more important contribution, or is known with higher confidence, than *e*_1 _and *e*_2_.

The ontology correlated clique score ((*C*, *E_c_*), *S*) is calculated as follows:

(4) $$Clique\text{\_}Score\left(\left(C,E_{c}\right),S\right)=\eta \left(S\right)\cdot |C|\cdot |S|\cdot Density\left(C,E_{c}\right)$$

where *S *is the common ontology attribute set of *C*. Based on equation (4), we can evaluate these ontology correlate cliques based on both topology structure and the similarity of the ontology attributes. Table 1 shows the statistics of the ontology correlated cliques in Figure 4.

**Table 1:** Statistics of the ontology correlated cliques in Figure 4.

*Clique*	*S*	*Density*	*η*(*S)*	*Clique_Score*
{P2, P5, P6}	{GS1}	0.667	0.625	1.251
{P2, P3, P5}	{GS1, GS2}	0.867	1	5.202
{P2, P3, P4, P5}	{GS1, GS2}	0.833	1	6.664

(***w*_1 _**= 0.6 and ***w*_2 _**= 0.4).

### Protein complex prediction from attributed PPI networks

Our method for predicting protein complexes from attributed PPI networks involves two phases. In the first phase, we use high-throughput PPI data, and biomedical literature PPI data with GO, to construct two attributed PPI networks. The relative contributions of the high-throughput and literature PPI data is weighted automatically for each network. In the second phase, high-throughput PPI data, and biomedical literature PPI data with GO, are used to construct two whole attributed PPI networks. Based on the relative contributions of the PPI data, we predict the protein complexes from the whole attributed PPI networks.

In the first phase, we construct two attributed PPI networks with GO slims annotations and each type PPI data in turn: one for high-throughput PPI data and GO slims annotations, and the other for biomedical literature PPI data and GO slims annotations. We set the initial contribution weight of PPI data as *w*_1 _= *w*_2 _= 0.5, and used the cliques mining algorithm [22] to enumerate all maximal cliques of three or more from the two attributed PPI networks in turn, and calculated the ontology attribute set for each maximal clique. The candidate clique set *Candidate *is comprised of all maximal ontology correlated cliques, which are generally overlapped. Maximal ontology correlated cliques in *Candidate *are ranked in descending order of clique score, denoted as {((*C*_1_, *E*_*c*1_), *S*_1_),((*C*_2_, *E*_*c*2_), *S*_2_),...,((*C*_*n*_, *E*_*cn*_), *S*_*n*_)}. The top ranked clique ((*C*_1_, *E*_*c*1_), *S*_1_) is then deleted from *Candidate *and inserted into the seed clique set *Seed*. To ensure the seed cliques non-overlapping, we use the same method [8] to remove or prune overlapping candidate cliques until the candidate clique set *Candidate *is empty. Two seed clique sets *Seed*_1 _and *Seed*_2 _are generated from the attributed PPI networks constructed using high-throughput PPI data and biomedical PPI data, respectively. The quality of seed clique set *Seed_i_*, generated from the attributed PPI networks constructed using *T_i _*type PPI data, indicated the value of *T_i _*type PPI data for protein complex prediction. Therefore, the average clique score can be used to evaluate the relative contributions of high-throughput experimental and literature PPI data. Based on equations (5) and (6), the contribution weight of *T_i _*type PPI data can be automatically calculated.

(5) $$C\text{\_}Degree\left(T_{i}\right)=\frac{\sum_{\left(\left(C_{j},E_{cj}\right),S_{j}\right)\in Seed_{Ti}}Clique\text{\_}Score\left(\left(C_{j},E_{cj}\right),S_{j}\right)\cdot |E_{cj}|}{\left|E_{Ti}\right|}$$

(6) $$wi=\frac{C\text{\_}Degree\left(T_{i}\right)}{\sum_{i=1}^{2}C\text{\_}Degree\left(T_{i}\right)}$$

where *Seed_Ti _*denotes the seed clique set generated from the attributed PPI networks constructed using *T_i _*type PPIs, and |*E_Ti_*| is the total number of *T_i _*type PPI data.

In the second phase, we firstly construct the whole attributed PPI networks using high-throughput PPI data, literature PPI data and GO slims annotations. Secondly, we use the contribution weight *w*_1 _and *w*_2 _computed in the first phase to weight the whole attributed PPI networks. Thirdly, we generate the seed clique set *Seed *from the whole attributed PPI networks using the method described above for the first phase. Finally, we augment the seed cliques by adding each close neighbor protein vertex one by one. The closeness score is used to measure how closely a protein vertex *P_k _*with ontology attribute set *S_k _*is connected to a seed clique ((*C_j_*, *E_cj_*), *S_j_*), where *P_k _*∉ *C_j_*. The closeness score of *P_k _*with respect to ((*C_j_*, *E_cj_*), *S_j_*) is defined as follows:

(7) $$Close\text{\_}Score\left(\left(P_{k},S_{k}\right),\left(\left(C_{j},E_{cj}\right),S_{j}\right)\right)=\frac{\left|S_{k}\cap S_{j}\right|}{\left|S_{j}\right|+1}\cdot \frac{\sum_{el_{\in }E_{p}}\sum_{T_{i}\in F_{e}\left(el\right)}w_{i}}{\left|C_{j}\right|}$$

where *E_p _*is the set of edges between *P_k _*and ((*C_j_*, *E_cj_*), *S_j_*). In equation (7), $\left|S_{k}\cap S_{j}\right|/)\left|S_{j}\right|+1$ gives the GO annotation attribute similarity and $\sum_{el_{\in }E_{p}}\sum_{T_{i}\in F_{e}\left(e_{i}\right)}w_{i}/)\left|C_{j}\right|$ calculates the topology connectivity between the protein vertex *P_k _*and the seed clique ((*C_j_*, *E_cj_*), *S_j_*). Therefore, the closeness score provides a reasonable combination of both annotation attribute similarity and topology connectivity. If the *Close *_ *Score*((*P_k_*, *S_k_)*,((*C_j_*, *E_cj_*), *S_j_*)) ≥ *extend *_ *thres*, then *P_k _*was added to the seed clique ((*C_j_*, *E_cj_*), *S_j_*). Therefore the final predicted protein complexes are generated by adding the close neighbor proteins to the seed cliques. Here, *extend_thres *is a predefined threshold and the value of *extend_thres *ranges from 0 to 1. The smaller the value of *extend_thres*, the more neighbor proteins are added to the seed clique. If the value of *extend_thres *is close to 1, only the closest neighbor proteins in both topology structure and biology attributes are added. The optimal value of *extend_thres *can usually be determined in preliminary experiments.

## Results and discussion

In this section, the datasets and evaluation metrics used in the experiments are described. The value of biomedical literature PPI data for protein complex prediction is then considered. The impact of the extend_thres parameter is assessed, along with the relative contributions of high-throughput and literature PPI data. Finally, our method is compared with current state-of-the-art protein complex prediction methods.

### Datasets and evaluation metrics

The two high-throughput PPI datasets used in our experiment are the Gavin dataset [3] and the Krogan dataset [23]. The Gavin dataset contains 1430 proteins and 6531 interactions, and the Krogan dataset contains 2675 proteins and 7080 interactions. The Biomedical literature data is a corpus of MEDLINE abstracts downloaded from PubMed. GO slim data is downloaded from http://www.yeastgenome.org. The benchmark protein complex dataset CYC2008 [24] includes 408 manually curated heterometric protein complexes for which experimental evidence has been reported.

To assess the quality of predicted protein complexes, we match generated complexes with the benchmark complex set CYC2008. Let *P*(*V_P_, E_P_*) be an predicted complex and *B*(*V_B_, E_B_*)) be a known complex. We define the neighborhood affinity score *NA(P,B) *between *P*(*V_P_, E_P_*)and *B*(*V_B_, E_B_*)) as follows:

(8) $$NA\left(P,B\right)=\frac{{\left|V_{P}\cap V_{B}\right|}^{2}}{\left|V_{P}\right|\times \left|V_{B}\right|}$$

In this experiment, we consider *P*(*V*_*P*_, *E*_*P*_) and *B*(*V*_*B*_, *E*_*B*_)) to match each other if *NA(P,B) *is larger than 0.2, which is the same as most methods for protein complex predication [12].

Precision, recall and *F-score *has been used to evaluate the performance of most previous protein complex prediction methods. Precision measures the fidelity of the predicted protein complex set. Recall quantifies the extent to which a predicted complex set captures the known complexes in the benchmark set. *F-score *provides a reasonable combination of both precision and recall, and can be used to evaluate the overall performance. Recently, sensitivity (Sn), positive predictive value (PPV) and accuracy (Acc) have also been used to evaluate protein complex prediction tools. Acc represents a tradeoff between Sn and PPV. The advantage of the geometric mean is that it yields a low score when either Sn or PPV are low. A high degree of accuracy thus requires a high performance for both criteria. These definitions have been described in detail by Li *et al*. [12]. To keep in line with most previous studies, we calculate precision, recall and *F-score *in this study, and also report Sn, PPV and Acc.

Most protein complex prediction studies reported to date have involved *Saccharomyces cerevisiae*. In this study, we download all *S. cerevisiae*-related abstracts from 1990-2012 from MEDLINE using PubMed. We use the user-defined kernel interface of the SVMLight package http://svmlight.joachims.org/ to implement the HSP kernel.

AImed, BioInfer, IEPA, HPRD50 and LLL are the five annotated PPI corpora that are most commonly used for PPI data extraction, and were used in this study to construct training datasets [19]. The results of the five training datasets (Table 2) include Annotated PPIs (the number of PPI data extracted from the annotated corpora) and Extracted PPIs (the number of PPI data extracted from the literature. Extracted PPI data found in only one of the Gavin or Krogan datasets is shown. BioInfer is the largest corpora among the five annotated PPI corpora, however only 1196 PPIs are extracted with the BioInfer training set. AImed extracts the most PPIs (2957), and LLL extracts 1871 PPIs despite only containing 300 annotated PPIs.

**Table 2:** PPI extraction results for five training corpora

Training dataset	Annotated PPIs	Extracted PPIs	Different from Gavin	Different from Krogan
AImed	5834	2957	2729	2659
BioInfer	9666	1196	1100	1073
IEPA	817	2223	2072	2039
HPRD50	433	2573	2390	2362
LLL	300	1871	1756	1722

Firstly, we apply COACH [9], CMC [8], Cluster ONE [11] and COAN [14] to the PPI data extracted from literature. *F*-score results on five literature PPI data are listed in Table 3. From Table 3, *it *can be seen that literature PPI data extracted by Aimed corpora achieves the highest *F*-score on four protein complex prediction methods. We next apply the four complex prediction methods to the hybrid PPI data that comprised high-throughput experimental and literature-extracted PPI data. The *F*-score on the Gavin dataset are listed in Table 4. "Gavin+AImed" denotes this data comprised Gavin PPI data and PPI data extracted from the literature using the AImed corpora training dataset. From Table 4 it can be seen that the *F*-scores of hybrid PPI data generally outperform those of the Gavin PPI data. For example, COACH and CMC are improved the *F*-score by 0.014 (from 0.406 to 0.42), and 0.038 (from 0.321 to 0.359) on the Gavin+AImed PPI data, respectively. In addition, we randomly add 1000, 2000 or 3000 interactions to the Gavin dataset. For each simulation, we perform 10 times randomization experiments and calculate the mean value and standard deviation. Table 4 shows that the *F*-scores achieved on the Gavin + random PPI data are inferior to on the Gavin PPI data.

**Table 3:** Results of PPI data extracted from biomedical literature.

	COACH	CMC	ClusterONE	COAN
AImed	0.1938	0.1395	0.1873	0.153
BioInfer	0.1233	0.1072	0.1578	0.1231
IEPA	0.1325	0.1015	0.1597	0.1422
HPRD50	0.1566	0.1175	0.1462	0.1396
LLL	0.1345	0.1103	0.1403	0.1325

**Table 4:** Results of Gavin PPI data and biomedical literature PPI data.

	COACH		CMC		ClusterONE		COAN	
	
	F	σ_F_	F	σ_F_	F	σ_F_	F	σ_F_
Gavin dataset	0.406	-	0.321	-	0.418	-	0.404	-
Gavin + random I	0.402	0.003	0.311	0.009	0.408	0.012	0.402	0.004
Gavin + random II	0.398	0.005	0.298	0.005	0.389	0.013	0.401	0.005
Gavin + random III	0.395	0.005	0.283	0.012	0.366	0.013	0.393	0.008
Gavin + Aimed	**0.42**	**-**	**0.359**	**-**	**0.429**	**-**	**0.428**	**-**
Gavin + BioInfer	0.414	-	0.329	-	0.415	-	0.413	-
Gavin + IEPA	0.406	-	0.342	-	0.427	-	0.409	-
Gavin +HPRD50	0.417	-	0.32	-	0.423	-	0.409	-
Gavin + LLL	0.41	-	0.337	-	0.411	-	0.419	-

Gavin + random I, Gavin + random II, and Gavin + random III show the results of randomly adding 1000, 2000 and 3000 interactions to the Gavin dataset, respectively. The highest *F*-score of each approach is shown in bold.

The *F*-scores for the Krogan dataset are listed in Table 5. Again, all four approaches achieve better performance on the hybrid PPI data. This suggests that integrating literature PPI data and high-throughput PPI data can effectively improve the performance of protein complex prediction.

**Table 5:** The results of Krogan PPI data and biomedical literature PPI data.

	COACH		CMC		ClusterONE		COAN	
	
	F	σ_F_	F	σ_F_	F	σ_F_	F	σ_F_
Krogan dataset	0.441	-	0.358	-	0.401	-	0.451	-
Krogan + random I	0.439	0.002	0.353	0.004	0.379	0.014	0.445	0.002
Krogan + random II	0.436	0.004	0.349	0.006	0.354	0.021	0.449	0.006
Krogan + random III	0.433	0.004	0.347	0.006	0.34	0.018	0.444	0.007
Krogan + Aimed	**0.457**	**-**	**0.411**	**-**	**0.417**	**-**	**0.464**	**-**
Krogan + BioInfer	0.453	-	0.366	-	0.405	-	0.458	-
Krogan + IEPA	0.444	-	0.398	-	0.393	-	0.453	-
Krogan + HPRD50	0.445	-	0.384	-	0.389	-	0.463	-
Krogan + LLL	0.454	-	0.393	-	0.404	-	0.453	-

Krogan + random I, Krogan + random II, and Krogan + random III show the results of randomly adding 1000, 2000 and 3000 PPIs to the Krogan dataset, respectively. The highest ***F***-score of each approach is shown in bold.

### The effect of *extend_thres*

We construct two attributed PPI networks to integrate high-throughput PPI data, biomedical literature PPI data and GO as described in the Section 2.2. Attributed PPI network I is constructed using GO, the Gavin dataset, and extracted PPI data using the AImed corpora as the training dataset. Attributed PPI network II is constructed using GO, the Krogan dataset, and extracted PPI data using the AImed corpora as the training dataset. To study the effect of the *extend_thres *parameter, we first evaluate our method on Attributed PPI network I. Our method proves sensitive to *extend_thres *between 0.05 and 0.6 (Table 6). The *F-score *performance ranges from 0.386 to 0.447., Precision, recall and F-score are 0.506, 0.314 and 0.387, respectively, when *extend_thres *= 0.05. This indicates that too many proteins are added to the seed cliques to construct complexes during the seed cliques augment phase, suggesting the value of *extend_thres *is too small. As *extend_thres *is increased, the number of proteins added decreased sharply. When *extend_thres *= 0.1, precision, recall and *F-score *improve significantly to 0.589, 0.36 and 0.447, respectively. When *extend_thres *is increased from 0.1 to 0.6, precision, recall and *F-score *all decrease. The closeness score calculated using equation (7) provides a reasonable combination of both annotation attribute similarity and topology connectivity. In order to maintain a closeness score larger than 0.6, the candidate proteins must have highly similar ontology attribute set and topology connectivity to seed cliques during the seed cliques augment phase. However, there are few such candidate proteins in the attributed PPI networks. Therefore, performance precision, recall and *F-score *are relatively unaffected when *extend_thres *varied between 0.6 and 1.0.

**Table 6:** The effect of extend_thres on protein complex prediction performance using Attributed PPI network I.

*Extend_thres*	P	R	F	Sn	PPV	Acc
0.05	0.506	0.314	0.387	**0.569**	0.389	0.471
0.1	**0.589**	**0.36**	**0.447**	0.521	0.541	**0.531**
0.2	0.55	0.341	0.421	0.451	0.611	0.525
0.3	0.524	0.321	0.398	0.39	0.653	0.505
0.4	0.515	0.311	0.388	0.349	0.677	0.486
0.5	0.506	0.314	0.387	0.332	0.699	0.482
0.6	0.502	0.314	0.386	0.331	**0.701**	0.482
0.7	0.502	0.314	0.386	0.331	**0.701**	0.482
0.8	0.502	0.314	0.386	0.331	**0.701**	0.482
0.9	0.502	0.314	0.386	0.331	**0.701**	0.482
1.0	0.502	0.314	0.386	0.331	**0.701**	0.482

F: *F-score*, P: precision, R: recall. The highest ***F***-score of each approach is shown in bold.

The Sn, PPV and Acc metrics are then evaluated. When *extend_thres *is changed from 0.05 to 0.6, PPV increased whereas Sn decreases. When *extend_thres *ranges between 0.6 and 1.0, Sn, PPV and Acc do not change appreciably (0.331, 0.701 and 0.482, respectively). This is due to more neighbor proteins being added to the seed cliques when *exend_thres *is low, resulting in the predicted complexes having better coverage of the benchmark dataset complexes, and improving the Sn metric. In contrast, only the closest neighbor proteins are added to the seed clique when *exend_thres *is high. This increases the likelihood of predicted complexes being true positives, and improves the PPV metric. Acc is defined as the geometric mean of Sn and PPV, which is potentially more comprehensive for evaluating performance. Similar to *F-score*, Acc is maximized (0.531) when *extend_thres *= 0.1.

We also evaluate the effects of *extend_thres *on Attributed PPI network II (Table 7; compare with Table 6). Again, the highest *F-score *(0.477) and Acc (0.551) are achieved when *extend_thres *= 0.1.

**Table 7:** The effect of extend_thres on protein complex prediction performance using Attributed PPI network II.

*Extend_thres*	P	R	F	Sn	PPV	Acc
0.05	0.571	0.316	0.407	0.581	0.413	0.49
0.1	**0.636**	**0.382**	**0.477**	0.525	0.576	**0.551**
0.2	0.599	0.367	0.457	0.447	0.647	0.538
0.3	0.567	0.365	0.444	0.389	0.702	0.523
0.4	0.559	0.355	0.434	0.348	0.72	0.501
0.5	0.551	0.35	0.428	0.339	0.732	0.498
0.6	0.551	0.348	0.426	0.336	**0.734**	0.497
0.7	0.551	0.348	0.426	0.336	**0.734**	0.497
0.8	0.551	0.348	0.426	0.336	**0.734**	0.497
0.9	0.551	0.348	0.426	0.336	**0.734**	0.497
1.0	0.551	0.348	0.426	0.336	**0.734**	0.497

F: *F-score*, P: precision, R: recall. The highest ***F***-score of each approach is shown in bold.

### The relative contributions of experimental and literature-extracted PPI data

We evaluate the relative contributions of high-throughput experimental and literature PPI data to protein complex prediction performance. Another strength of our method is that it automatically computes contribution weights. The statistics of the contributions of literature data are listed in Table 8. In Attributed PPI network I, high-throughput and literature PPI data contribute weights of 0.59 and 0.41, respectively. In Attributed PPI network II, these are 0.55 and 0.45, respectively. Furthermore, we evaluate the effect of weight mechanism on these networks (Table 9). When no weight mechanism is used, equal weight is given to high-throughput and literature PPI data. The weight mechanism improves F-score by 0.025 and 0.014 on Attributed PPI networks I and II, respectively.

**Table 8:** The contribution weight of high-throughput experimental and literature PPI data using Attributed PPI networks for protein complex prediction.

		High-throughput	Literature
Attributed networks I	PPIs	0.59	0.41
	Weight	6351	2957

Attributed networks II	PPIs	7080	2957
	Weight	0.55	0.45

Extend_thres = 0.1.

**Table 9:** Performance comparison of the weight mechanism.

	P	R	F	Sn	PPV	Acc
Weight I	0.589	0.36	0.447	0.521	0.541	0.531
No weight I	0.556	0.341	0.422	0.52	0.526	0.523
Weight II	0.636	0.382	0.477	0.525	0.576	0.551
No weight II	0.623	0.368	0.463	0.523	0.567	0.544

Weight I and Weight II denote the performance using the weight mechanism on attributed PPI networks I and II, respectively. No weight I and No weight II denote the performance without the weight mechanism F: *F-score*, P: precision, R: recall.

### Comparison with other protein complex prediction methods

We compare our method with the following established leading protein complex prediction methods: Cluster ONE [11], COACH [9], CMC [8], HUNTER [25], MCL [5] and MCODE [4] (Table 10).

**Table 10:** Performance comparison with other protein complex prediction methods.

PPIN	Methods	#Complexes	P	R	F	Sn	PPV	Acc
Attr. PPIN I	Our method (BP,MF,CC)	231	0.589	**0.36**	**0.447**	0.521	0.541	0.531
	
	Our method (BP,MF)	182	0.659	0.326	0.436	0.471	0.571	0.518

	ClusterONE	199	0.568	0.331	0.418	0.468	**0.609**	**0.534**
	COACH	326	0.525	0.333	0.406	0.44	0.547	0.49
	CMC	120	0.608	0.218	0.321	0.371	0.606	0.474
Gavin PPIN	HUNTER	69	**0.87**	0.206	0.333	0.386	0.508	0.443
	MCL	103	0.718	0.245	0.366	**0.53**	0.489	0.509
	MCODE	70	0.739	0.154	0.255	0.283	0.519	0.384

Attr. PPIN II	Our method (BP,MF,CC)	247	0.636	0.382	**0.477**	0.525	0.576	0.551
	
	Our method (BP,MF)	206	0.679	0.348	0.46	0.477	0.578	0.525

	ClusterONE	464	0.375	**0.431**	0.401	0.523	**0.655**	**0.585**
	COACH	345	0.617	0.343	0.441	0.432	0.544	0.485
Krogan PPIN	CMC	111	0.748	0.235	0.358	0.381	0.589	0.474
	HUNTER	74	**0.865**	0.199	0.323	0.374	0.569	0.462
	MCL	309	0.291	0.245	0.266	**0.57**	0.396	0.475
	MCODE	72	0.75	0.159	0.263	0.27	0.552	0.386

#Complexes refers to the number of predicted complexes. F: *F-score*, P: precision, R: recall. The highest *F*-score of each approach is shown in bold.

GO provides GO terms or slims to describe gene product characteristics in three different aspects, including Biological Process (BP), Molecular Function (MF) and Cellular Component (CC). In the GO data, Some of CC attributes are directly pertinent to protein complex. Firstly, we evaluate the effect of CC attributes of GO data to our method. In the Table 10, "Our method (MF, BP, CC)" and "Our method (MF, BP)" denote our method performed on whole GO slim data, and the GO slim data which removes CC attributes set, respectively. From Table 10, it can be seen that the F-score reduces 0.011 and 0.017 on Attributed PPI network I and II, respectively, when CC attributes set is removed from GO slim data.

Secondly, we compare our method using Attributed PPI network I with Cluster ONE, COACH, CMC, HUNTER, MCL and MCODE using the Gavin PPI network. Our method achieves an F-score of 0.447, which is significantly superior to the other methods (Table 10). Cluster ONE achieves the highest Acc of 0.534. It is worth noting that COACH predicts 326 complexes, which is much more than other methods. In contrast, HUNTER only predicts 69 complexes, albeit with the highest precision of 0.87 and a low recall of 0.206. MCL predicts 103 complexes, and achieves the highest Sn of 0.53.

Finally, we compare our method using Attributed PPI network II with the other methods using the Krogan PPI network. From Table 10, it can be seen that our method also achieves the highest F-score of 0.477. Cluster ONE achieves the highest Acc of 0.585. HUNTER and MCL achieve the highest precision (0.865) and Sn (0.57), respectively.

In summary, our method can integrate high-throughput experimental PPI data, biomedical literature PPI data, and GO by constructing attributed PPI networks. This approach outperforms existing protein complex prediction tools.

## Conclusions

We exploite the natural language processing technique to extract PPI data from the biomedical literature and integrate this data with high-throughput experimental PPI data and GO to construct attributed PPI networks. Using these networks, we develope a novel method for protein complex prediction that automatically calculate the relative contributions of experimental and literature data. This approach outperforms established leading protein complex prediction tools. In the future, we intend to incorporate a post-processing phase and make even better use of literature data extraction to further improve protein complex prediction performance.

## Competing interests

The authors declare that they have no competing interests.

## Declarations

This work is supported by grant from the Natural Science Foundation of China (No. 61300088, 61340020 and 61272373), the Fundamental Research Funds for the Central Universities (No. DUT14QY44).

This article has been published as part of *BMC Genomics *Volume 16 Supplement 2, 2015: Selected articles from the Thirteenth Asia Pacific Bioinformatics Conference (APBC 2015): Genomics. The full contents of the supplement are available online at http://www.biomedcentral.com/bmcgenomics/supplements/16/S2

## References

[B1] Li M Chen J-e Wang J-x Hu B Chen G Modifying the DPClus algorithm for identifying protein complexes based on new topological structures BMC bioinformatics 2008 9 1 398 10.1186/1471-2105-9-398 18816408 PMC2570695

[B2] Rajagopala SV Sikorski P Kumar A Mosca R The binary protein-protein interaction landscape of Escherichia coli 2014 32 3 2851 290 10.1038/nbt.2831 24561554 PMC4123855

[B3] Gavin A-C Bösche M Krause R Grandi P Marzioch M Bauer A Schultz J Rick JM Michon A-M Cruciat C-M Functional organization of the yeast proteome by systematic analysis of protein complexes Nature 2002 415 6868 141 147 10.1038/415141a 11805826

[B4] Bader GD Hogue CW An automated method for finding molecular complexes in large protein interaction networks BMC bioinformatics 2003 4 1 2 10.1186/1471-2105-4-2 12525261 PMC149346

[B5] Srihari S Ning K Leong HW MCL-CAw: a refinement of MCL for detecting yeast complexes from weighted PPI networks by incorporating core-attachment structure BMC bioinformatics 2010 11 1 504 10.1186/1471-2105-11-504 20939868 PMC2965181

[B6] Adamcsek B Palla G Farkas IJ Derényi I Vicsek T CFinder: locating cliques and overlapping modules in biological networks Bioinformatics 2006 22 8 1021 1023 10.1093/bioinformatics/btl039 16473872

[B7] Palla G Derényi I Farkas I Vicsek T Uncovering the overlapping community structure of complex networks in nature and society Nature 2005 435 7043 814 818 10.1038/nature03607 15944704

[B8] Liu G Wong L Chua HN Complex discovery from weighted PPI networks Bioinformatics 2009 25 15 1891 1897 10.1093/bioinformatics/btp311 19435747

[B9] Wu M Li X Kwoh C-K Ng S-K A core-attachment based method to detect protein complexes in PPI networks BMC bioinformatics 2009 10 1 169 10.1186/1471-2105-10-169 19486541 PMC2701950

[B10] Gavin A-C Aloy P Grandi P Krause R Boesche M Marzioch M Rau C Jensen LJ Bastuck S Dümpelfeld B Proteome survey reveals modularity of the yeast cell machinery Nature 2006 440 7084 631 636 10.1038/nature04532 16429126

[B11] Nepusz T Yu H Paccanaro A Detecting overlapping protein complexes in protein-protein interaction networks Nature methods 2012 9 5 471 472 10.1038/nmeth.1938 22426491 PMC3543700

[B12] Li X Wu M Kwoh C-K Ng S-K Computational approaches for detecting protein complexes from protein interaction networks: a survey BMC genomics 2010 11 Suppl 1 S3 10.1186/1471-2164-11-S1-S3 20158874 PMC2822531

[B13] Feng J Jiang R Jiang T A max-flow-based approach to the identification of protein complexes using protein interaction and microarray data IEEE/ACM Transactions on Computational Biology and Bioinformatics (TCBB) 2011 8 3 621 634 20733237 10.1109/TCBB.2010.78

[B14] Zhang Y Lin H Yang Z Wang J Construction of Ontology Augmented Networks for Protein Complex Prediction PloS one 2013 8 5 e62077 10.1371/journal.pone.0062077 23650509 PMC3641129

[B15] Zhang Y Lin H Yang Z Wang J Li Y Hash Subgraph Pairwise Kernel for Protein-Protein Interaction Extraction IEEE/ACM Transactions on Computational Biology and Bioinformatics (TCBB) 2012 9 4 1190 1202 22595237 10.1109/TCBB.2012.50

[B16] Yang Z Zhao Z Li Y Hu Y Lin H A Protein Interaction Extraction and Visualization System for Biomedical Literature IEEE Transactions on Nanobioscience 2013 12 3 173 181 10.1109/TNB.2013.2263837 23974658

[B17] Li Y Lin H Yang Z Incorporating rich background knowledge for gene named entity classification and recognition BMC bioinformatics 2009 10 1 223 10.1186/1471-2105-10-223 19615051 PMC2725142

[B18] Kim J-D Ohta T Tateisi Y Tsujii Ji GENIA corpus--a semantically annotated corpus for bio-textmining Bioinformatics 2003 19 suppl 1 i180 i182 10.1093/bioinformatics/btg1023 12855455

[B19] Pyysalo S Airola A Heimonen J Björne J Ginter F Salakoski T Comparative analysis of five protein-protein interaction corpora BMC bioinformatics 2008 9 Suppl 3 S6 10.1186/1471-2105-9-S3-S6 18426551 PMC2349296

[B20] Ashburner M Ball CA Blake JA Botstein D Butler H Cherry JM Davis AP Dolinski K Dwight SS Eppig JT Gene Ontology: tool for the unification of biology Nature genetics 2000 25 1 25 29 10.1038/75556 10802651 PMC3037419

[B21] Silva A Meira W Jr Zaki MJ Mining attribute-structure correlated patterns in large attributed graphs Proceedings of the VLDB Endowment 2012 5 5 466 477 10.14778/2140436.2140443

[B22] Tomita E Tanaka A Takahashi H The worst-case time complexity for generating all maximal cliques and computational experiments Theoretical Computer Science 2006 363 1 28 42 10.1016/j.tcs.2006.06.015

[B23] Krogan NJ Cagney G Yu H Zhong G Guo X Ignatchenko A Li J Pu S Datta N Tikuisis AP Global landscape of protein complexes in the yeast Saccharomyces cerevisiae Nature 2006 440 7084 637 643 10.1038/nature04670 16554755

[B24] Pu S Wong J Turner B Cho E Wodak SJ Up-to-date catalogues of yeast protein complexes Nucleic acids research 2009 37 3 825 831 10.1093/nar/gkn1005 19095691 PMC2647312

[B25] Chin C-H Chen S-H Ho C-W Ko M-T Lin C-Y A hub-attachment based method to detect functional modules from confidence-scored protein interactions and expression profiles BMC bioinformatics 2010 11 Suppl 1 S25 10.1186/1471-2105-11-S1-S25 20122197 PMC3009496

